# New methods for indexing multi-lattice diffraction data

**DOI:** 10.1107/S1399004714017039

**Published:** 2014-09-27

**Authors:** Richard J. Gildea, David G. Waterman, James M. Parkhurst, Danny Axford, Geoff Sutton, David I. Stuart, Nicholas K. Sauter, Gwyndaf Evans, Graeme Winter

**Affiliations:** a Diamond Light Source Ltd, Harwell Science and Innovation Campus, Didcot OX11 0DE, England; b STFC Rutherford Appleton Laboratory, Didcot OX11 0QX, England; c CCP4, Research Complex at Harwell, Rutherford Appleton Laboratory, Didcot OX11 0FA, England; d Division of Structural Biology, The Wellcome Trust Centre for Human Genetics, University of Oxford, Oxford OX3 7BN, England; e Physical Biosciences Division, Lawrence Berkeley National Laboratory, Berkeley, CA 94720, USA

**Keywords:** indexing, multi-lattice data

## Abstract

A new indexing method is presented which is capable of indexing multiple crystal lattices from narrow wedges of diffraction data. The method takes advantage of a simplification of Fourier transform-based methods that is applicable when the unit-cell dimensions are known *a priori*. The efficacy of this method is demonstrated with both semi-synthetic multi-lattice data and real multi-lattice data recorded from crystals of ∼1 µm in size, where it is shown that up to six lattices can be successfully indexed and subsequently integrated from a 1° wedge of data. Analysis is presented which shows that improvements in data-quality indicators can be obtained through accurate identification and rejection of overlapping reflections prior to scaling.

## Abstract 2

A new indexing method is presented which is capable of indexing multiple crystal lattices from narrow wedges of data. The efficacy of this method is demonstrated with both semi-synthetic multi-lattice data and real multi-lattice data recorded from microcrystals of ∼1 µm in size.

## 1. Introduction   

A fundamental limitation of conventional macromolecular crystallography is the necessity of obtaining one or more crystals of sufficient size and quality to record a reasonably complete data set. The development of microfocus beamlines has allowed data to be collected from smaller crystals than ever before [see the recent reviews of the history and capabilities of microfocus beamlines by Evans *et al.* (2011 ▶) and Smith *et al.* (2012 ▶)]. Frequently, particularly in the cases of viruses and membrane proteins, only small, poor-quality crystals may be available and it may only be possible to collect a highly incomplete data set over a small oscillation range for each individual crystal before the diffraction quality is affected by radiation damage.

While an individual crystal may only give an incomplete partial data set, a complete data set may be obtained by merging data from many tens or hundreds of crystals (Grimes *et al.*, 1998 ▶; Wang *et al.*, 2012 ▶; Hanson *et al.*, 2012 ▶) (although in certain circumstances very incomplete data sets may suffice; see, for example, Hadfield *et al.*, 1995 ▶). The advent of serial femtosecond crystallography using X-ray free-electron lasers (XFELs) has recently encouraged further interest in the development of serial crystallography using synchrotrons (Gati *et al.*, 2014 ▶; Rossmann, 2014 ▶; Stellato *et al.*, 2014 ▶).

For crystals as small as a few micrometres in size it may not be possible to resolve individual crystals using the beamline on-axis viewing system, in which case grid-scan analysis (Song *et al.*, 2007 ▶; Cherezov *et al.*, 2009 ▶; Bowler *et al.*, 2010 ▶; Aishima *et al.*, 2010 ▶; Axford *et al.*, 2012 ▶) may be necessary to identify sample locations prior to data collection. Such grid scans usually score the diffraction quality by the number and intensity of diffraction spots, suggesting positions where these are maximized. While this is generally a reliable procedure, in cases where the samples are substantially smaller than the beam it is likely that positions will be selected where multiple samples are illuminated, such that multiple independent diffraction patterns will be visible in the resulting data. Unfortunately, indexing methods in the more commonly used integration packages can become unreliable when multiple similarly strong lattices are visible in narrow wedges of data.

In *XDS* (Kabsch, 2010*b* ▶) indexing generally works well when there is a single dominant lattice (Kabsch, 1993 ▶); however, it may work less well or not at all when two or more equally strong lattices are present. Within *MOSFLM* (Leslie & Powell, 2007 ▶) it is now possible to index as many as four independent lattices (Powell *et al.*, 2013 ▶); however, this requires the use of at least two images well spaced in rotation and may also require careful adjustment of parameters. For the multi-lattice indexing in *LABELIT* (Sauter & Poon, 2010 ▶) it is assumed that one main lattice may be assigned which identifies (by default) at least 40% of the reflections. Paithankar *et al.* (2011 ▶) described the application of the *GrainSpotter* program (Sørensen *et al.*, 2012 ▶; Schmidt, 2014 ▶) to multi-lattice macromolecular crystallography, but *GrainSpotter* does not currently appear to be in widespread use within the macromolecular crystallography community.

Here, we present a new indexing method to address this challenge of indexing multiple similarly strong lattices within narrow wedges of data. The algorithms for these methods have been developed within the *DIALS* framework (Waterman *et al.*, 2013 ▶), which builds on *cctbx* (Grosse-Kunstleve *et al.*, 2002 ▶) and *dxtbx* (Parkhurst *et al.*, 2014 ▶) to offer tools for the analysis of X-ray diffraction data. In the context of indexing diffraction patterns, this offers spot finding, refinement, handling of Bravais lattice constraints (Grosse-Kunstleve *et al.*, 2004 ▶) and tools for exporting the results to, for example, *XDS*. The methods presented here are therefore implemented in the program *dials.index*.

## 2. Notation   

For clarity, the following notation will be used in this manuscript for the mathematical operations. A more complete description, including a discussion of the various coordinate frames used, may be found in Appendix *A* [App appa] and also in the description by Parkhurst *et al.* (2014 ▶) of the experimental models used by *dxtbx*. Throughout this manuscript we use the term ‘sweep’ to refer to a contiguous sequence of rotation images measured with a constant wavelength, distance and dose per image. A ‘wedge’ typically refers to a small sweep, *i.e.* one that samples a small part of reciprocal space.

λ: X-ray wavelength.

**h**: Miller indices *h*, *k*, *l*; **h**′ is its real-valued approximation.

**U**, **B** and **A**: crystal orientation matrix, reciprocal-space orthogonalization matrix and setting matrix, respectively, where **A** = **UB** = (a* b* c*) and a*, b*, c* are the reciprocal-space unit-cell vectors.

**A** ^−1^: indexing matrix, where **A** ^−1^ = (**a** **b** **c**)^T^ and **a**, **b**, **c** are the real-space unit-cell vectors.

ϕ: rotation angle around the goniostat rotation axis.

**R**: goniostat rotation matrix.

**d**_*x*_, **d**_*y*_: basis vectors for coordinates in the detector plane.

**d**_0_: vector from the origin of the laboratory frame to the origin of coordinates for the detector plane.

**D**: detector projection matrix, where **D** = **d** ^−1^ = (**d**_*x*_ **d**_*y*_ **d**_0_)^−1^ (Bricogne, 1987 ▶).

*x*_px_, *y*_px_ and *x*_mm_, *y*_mm_: detector pixel coordinates and coordinates in millimetres in a virtual detector plane, respectively.

**v**: virtual detector coordinates, where **v** = (*x*_mm_, *y*_mm_, 1).

**s**_0_, **s**_1_: incident and scattered beam vector.

**r**_ϕ_: reciprocal-lattice vector on the surface of the Ewald sphere at rotation angle ϕ, where **r**_ϕ_ = **RAh**.

**r**: reciprocal-lattice vector in Cartesian reciprocal space (*i.e.* fixed with respect to the laboratory frame), where **r** = **Ah**.

## 3. Methods   

Indexing methods conventionally take a list of spot centroid positions (whether three-dimensional centroids or two-dimensional image centroids and frame numbers) and some description of the experimental geometry to (i) convert the spot positions to the laboratory frame, (ii) convert these positions to the corresponding set of reciprocal-space vectors {**r**_*j*_} and (iii) analyse the set of vectors {**r**_*j*_} for periodicity and hence find the reciprocal-lattice basis vectors and the corresponding set of integer Miller indices {**h**_*j*_}. In mathematical terms the first two steps are common to all indexing methods as follows (Pflugrath, 1997 ▶).

A sequence of diffraction images are analysed to find a list of candidate Bragg reflections using a spotfinding routine. This returns a list of spot centroids in the form of *x*_px_, *y*_px_ pixel coordinates in the detector plane and image numbers (which may be non-integral for three-dimensional spotfinding), which are then mapped to *x*_mm_, *y*_mm_ positions in the detector coordinate system (1) and a rotation angle ϕ. Consequently, these are mapped onto the surface of the Ewald sphere to give the scattered beam wavevector, **s**_1_, normalized to length 1/λ (2–4 [Disp-formula fd3] [Disp-formula fd4]), where λ is the wavelength, such that the end point of the vector is on the surface of the Ewald sphere with radius 1/λ. The reciprocal-lattice vector in diffracting condition, **r**_ϕ_, is obtained as the difference between the diffracted wavevector **s**_1_ and the incident beam vector **s**_0_ (5). The reciprocal-lattice vector in Cartesian reciprocal space, **r**, is obtained by rotating the vector **r**_ϕ_ by the angle −ϕ about the vector defined by the rotation axis of the goniometer (6).
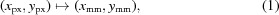


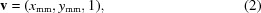


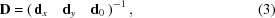


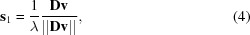


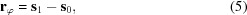


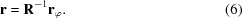
Analysis of the set of reciprocal-lattice vectors {**r**_*j*_} to determine the basis vectors may use a variety of algorithms. In *XDS* (Kabsch, 1988 ▶) the set of short difference vectors {**r**_*j*_ − **r**_*k*_} are calculated to build up low-order multiples of lattice vectors on a histogram, which is subsequently analysed to determine a unique basis. Other methods rely on the Fourier transform relationship between real and reciprocal space to provide a route for simultaneously determining both the unit-cell and crystal-orientation parameters from a set of observed spot centroids (Bricogne, 1986 ▶; Otwinowski & Minor, 1997 ▶; Steller *et al.*, 1997 ▶; Campbell, 1998 ▶). Methods have been developed utilizing both one-dimensional (Steller *et al.*, 1997 ▶; Powell, 1999 ▶; Sauter *et al.*, 2004 ▶) and three-dimensional (Campbell, 1998 ▶; Otwinowski *et al.*, 2012 ▶) fast Fourier transforms (FFT) to identify the likely directions and magnitudes of the reciprocal-lattice vectors.

These published Fourier methods utilize the knowledge that the maxima of the function 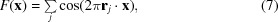
where **x** represents a point in direct space, are the solutions giving integer triples, **h**_*j*_, to the set of equations 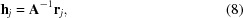
where 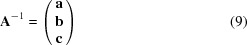
and **a**, **b** and **c** are the initially unknown unit-cell basis vectors. The vectors **x** that give the maximum values of *F*(**x**) correspond, therefore, to these real-space unit-cell basis vectors or some linear combination thereof. A three-dimensional fast Fourier transform may be used to calculate this function on a relatively coarse three-dimensional uniform grid, which is then searched to find the approximate maxima of (7) (Campbell, 1998 ▶; Otwinowski *et al.*, 2012 ▶). Alternatively, the maxima may be found by carrying out a series of one-dimensional FFTs after projecting the reciprocal basis vectors onto various directions covering a hemisphere of reciprocal space (Steller *et al.*, 1997 ▶).

The methods described above simultaneously determine both the direction and magnitude of the basis vectors. However, if the unit-cell dimensions are known then the magnitudes of the basis vectors are also known, leaving only the directions of the basis vectors to be determined. From the knowledge of the magnitude of the basis vectors, we know that each local maximum of (7) must lie on the surface of a sphere whose radius is determined by the magnitude of the basis vectors. Therefore, we propose to perform a two-dimensional, rather than a three-dimensional, search for maxima of (7) by varying the direction of **x** only, *i.e.*

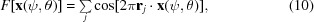
where 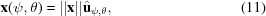
||**x**|| is set equal to the length of one of the real-space unit-cell vectors and 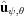 defines a unit vector with spherical coordinates ψ, θ. The search directions ψ, θ are chosen to be evenly spaced within a hemisphere, using a method similar to that described by Steller *et al.* (1997 ▶). The resulting set of vectors are sorted by decreasing value of *F*(**x**), and vectors that are approximately collinear with a vector higher in the list are eliminated. The top 30 vectors in this reduced list are analysed to find suitable combinations of basis vectors which are consistent (within user-defined relative length and absolute angular tolerances) with the known unit cell (Hattne *et al.*, 2014 ▶). This gives a set of candidate crystal setting matrices which are further analysed to choose the one which is most consistent with the set of observed centroids, *i.e.* the one which indexes as many observed centroids as possible. Although this relatively simple metric appears to work well in this study, work is ongoing to devise a more robust metric that takes into account the quality of the fit between the calculated and predicted centroids, such as that described by Sauter *et al.* (2004 ▶). The unit-cell dimensions of the primitive setting of the unit cell are used in the search for the initial set of candidate basis vectors, although the algorithm should be equally applicable in the case of the reduced basis, reference setting or some other nonstandard setting.

Each reciprocal-lattice vector is then expressed in terms of the reciprocal basis vectors according to 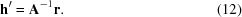
The nonintegral Miller indices **h**′ are rounded to give the integer Miller indices **h**. Only those reflections are used where the norm of the difference between the integer and real-valued Miller indices, *i.e.* ||**h**′ − **h**||, is less than some tolerance (in this work a tolerance of 0.3 was used).

The unit-cell and crystal-orientation parameters are then refined using the positions of the indexed reflections (§3.3). Once refinement has converged, any remaining unindexed reflections may be analysed for further lattices. In subsequent iterations, joint refinement of the crystal lattices is performed. This process may be repeated until either an insignificant number of unindexed reflections remain or no further lattices can be identified. If at any stage refinement does not converge, the most recently identified lattice is discarded and only those lattices which were refined successfully are reported.

### 3.1. Assigning indices to reflections in the presence of multiple lattices   

Initially, each reflection is assigned a potential Miller index as described above for the case of a single lattice. The reflection is assigned to the lattice that gives the Miller index with the smallest norm ||**h**′ − **h**||. A further check is made to ensure that two reflections are not assigned to the same lattice with the same Miller index. If this is the case, then the one that gives the smallest value of the norm ||**h**′ − **h**|| is used and the remaining reflections are rejected as outliers.

### 3.2. Spotfinding   

Spotfinding was performed using *dials.find_spots* (unpublished work), which is based on the algorithms described by Kabsch (2010*a* ▶). This determines spot centroids in three dimensions, as well as estimates of the centroid variances, which are a valuable input to the refinement step (§[Sec sec3.2]3.2).

*dials.find_spots* provides options to filter the initial list of strong spots based on the minimum number of contiguous pixels within the spot, the minimum and maximum resolution limits, the maximum peak-to-centroid separation (for example to reject split peaks) and the rejection of spots that are close to an ice ring or within an untrusted region of the detector (for example behind the beamstop shadow).

### 3.3. Refinement   

Refinement was performed with *dials.refine* (unpublished work) which includes a completely general approach to the refinement of the experimental geometry. This refinement minimizes *via* weighted least squares the discrepancy between the observed spot centroids and the central impacts calculated from the current model of the unit cell, crystal orientation, beam direction and detector position and orientation. Parameters that affect the shape of the spot, such as the mosaic spread, are not refined at this stage.

### 3.4. Outlier rejection   

Even for the case of a single lattice, outlier rejection can be important for accurate refinement of the crystal and experimental parameters. In the presence of multiple lattices, outlier rejection becomes critical for correctly assigning reflections to the separate lattices (Sauter & Poon, 2010 ▶). While Sauter & Poon (2010 ▶) propose a more elaborate statistical treatment of outliers, in this work we simply provide user-configurable parameters to control the maximum acceptable deviations between the observed and calculated spot position in *x* and *y* in the detector frame and in the rotation angle ϕ. This is similar to the behaviour of the equivalent *XDS* parameters (MAXIMUM_ERROR_OF_SPOT_POSITION= and MAXIMUM_ERROR_OF_SPINDLE_POSITION=), which have default values of three pixels and 2°, respectively (http://xds.mpimf-heidelberg.mpg.de/html_doc/xds_parameters.html).

### 3.5. The importance of accurate experimental geometry for indexing   

The mapping of the positions of diffraction maxima from image to reciprocal space is necessarily sensitive to the accuracy of the experimental description. For many single-lattice data sets, particularly spanning many degrees of rotation, assumptions may be made about the initial experimental geometry, for example assuming that the beam is perpendicular to the rotation axis and that this axis is coincident with the fast or slow direction on the detector. In most cases the deviation from these assumptions will be small and well within the radius of convergence of the indexing algorithms.

Hattne *et al.* (2014 ▶) demonstrated using XFEL still shots that poorly determined detector geometry can adversely affect both the indexing success rate and the quality of the integrated data, particularly at high resolution. Similarly, in the case of narrow wedges of synchrotron rotation data there is much less unique information to use in the refinement of the geometry. Combined with the presence of multiple lattices, which make outlier rejection more challenging, indexing methods become much less tolerant of errors in the recorded geometry. This is ideally addressed by (i) storing an accurate model of the experimental geometry in the image headers or (ii) having a good-quality and complete rotation data set recorded from a test crystal using the same experimental geometry. In many cases the latter of these is more easily achieved as a user, so *dials.index* allows the input of this refined geometric information from a previous processing run.

In some cases it may be found after the experiment is complete that the geometry recorded in the image headers is insufficiently accurate. In this situation it may be necessary to make some assumptions about the initial experimental geometry as above and attempt to discover, for example, a more accurate estimate of the beam centre (Sauter *et al.*, 2004 ▶). Of course, once the initial indexing is successful full refinement may proceed as described above.

### 3.6. Integration with *XDS*   

The resulting crystal and experimental geometry parameters were exported in *XDS* format and the separate lattices were integrated individually using *XDS*. Standard *XDS* practices were followed including, for example, running the *INTEGRATE* step a second time using the *GXPARM.XDS* output by the *CORRECT* step and the refined values for beam divergence and mosaicity as input (http://strucbio.biologie.uni-konstanz.de/xdswiki/index.php/Optimisation). 

### 3.7. Identification of overlapping reflections   

Ideally, overlapping reflections would be identified prior to integration in order that they can be excluded during determination of the spot profile model and taken into account during calculation of the background around each reflection. As *XDS* does not currently support integration of multiple lattices, analysis of overlapping reflections is performed after integration of the individual lattices with *XDS*, allowing overlapping reflections to be excluded from subsequent scaling.

In order to identify overlapping reflections, the extent of the reflections in detector/rotation space is first calculated as a bounding box that fully encloses each reflection’s peak region. In *DIALS*, the bounding box is created using a profile model as used in *XDS* and described by Kabsch (2010*a* ▶): the *XDS* σ_b_ and σ_m_ parameters are used to specify the size of each reflection on the detector and in rotation, respectively. The profile model assumes a Gaussian spot profile in a reciprocal-space coordinate system local to each reflection. The extent of the spot in this coordinate system is taken as *N*_σ_ standard deviations from the origin. The bounding box is then calculated by mapping the spot profile back into detector/rotation space and finding a three-dimensional box that fully encloses it. A mask is created for each reflection that specifies, for each pixel in the bounding-box region, which pixels are part of the peak and which are background. Overlapping reflections are then found in a two-stage procedure: the bounding boxes are first processed to extract a list of pairs of potentially overlapping reflections and these pairs are then checked to determine whether the peak regions overlap.

A space-partitioning algorithm is used to extract a list of overlapping bounding boxes. The algorithm uses a *k*-d tree to recursively partition the space along each dimension and query the number of objects intersecting a given range. Since the list of bounding boxes is used both to construct the tree and to provide the list of query ranges, an optimization is performed to allow these steps to be performed in a single pass. The algorithm has a time complexity of *O*(*N*log*N*), where *N* is the number of reflections. Each pair of potential overlaps is then analysed to determine whether their peak regions overlap. This is performed by iterating over the pixels in the intersection between the bounding boxes of two reflections; a pair of reflections which contains one or more pixels that are labelled as peak in both reflections are marked as overlapping.

### 3.8. Resolution of indexing ambiguities   

For several space groups, the Bravais lattice contains two or four symmetry elements that are not in the space group, resulting in alternative indexing possibilities (Dauter, 1999 ▶). For these space groups it is necessary to ensure that indexing is consistent across all crystals when merging data from multiple crystals to form a single data set. Programs such as *POINTLESS* (Evans, 2006 ▶) can typically resolve indexing ambiguities by comparing data from each crystal against a reference data set (which may be one of the data sets being scaled together). However, as the wedges of data being scaled together become narrower (*e.g.* 1°) this approach may no longer work reliably. If available, a more complete but low-resolution data set may be used as a reference, or calculated structure factors may be used if the structure is already known.

Brehm & Diederichs (2014 ▶) introduced an elegant way of breaking the indexing ambiguity in XFEL data sets, which may comprise many thousands to hundreds of thousands of very incomplete partial data sets taken from still shots. The approach rests upon a comparison of pairs of images. Regardless of the fact that still shots from two randomly oriented crystals will share only a few common Miller indices, the correlation coefficient between those shared structure-factor intensities will be highest if the two images have been indexed with the same sense. An implementation of algorithm 2 of Brehm & Diederichs (2014 ▶) was developed in the context of *cctbx.xfel* (Sauter *et al.*, 2013 ▶), and in §[Sec sec4.2]4.2 we demonstrate the application of the algorithm to synchrotron data in space group *I*23.

## 4. Results and discussion   

### 4.1. Semi-synthetic multi-lattice data sets   

Assessing the accuracy of the indexing method with multiple lattices present is straightforward if the correct orientation matrices are known *a priori*. To meet this requirement, semi-synthetic multi-lattice data sets were created by the pixel-wise addition of small wedges of data recorded from a crystal of bovine pancreatic trypsin (∼0.1 × 0.1 × 0.2 mm in size) on beamline I04 at Diamond Light Source at arbitrary ϕ and κ offsets of a mini-kappa gonio­meter. Each wedge of data was recorded over a total range of 10° with 0.1° and 0.1 s per image, with a relatively low transmission (5%) to minimize the effects of radiation damage. The data were recorded at a wavelength of 0.97949 Å at a distance of 214 mm on a PILATUS2 6M detector. A total of 12 data sets were recorded (*a*–*l*) and combined to create data sets with two, three, four and six lattices visible by adding the intensity at pixel *x*, *y* on image *z* from, for example, data sets *a*, *e* and *i* for the pixel at *x*, *y* on image *z* for data set *aei*. For a given number of lattices each of the original image sets was used only once. For each and every permutation of data sets, the orientations of all crystals were successfully identified and refined using the methods described in this paper. Each lattice was integrated individually using *XDS* (Kabsch, 2010*b* ▶) before the 12 integrated partial data sets were scaled together using *AIMLESS* (Evans & Murshudov, 2013 ▶). It is important to note that by default *AIMLESS* adjusts the intensity standard deviations automatically as σ′(*I*) = SdFac[σ(*I*)^2^ + SdB × *I* + (SdAdd × *I*)^2^]^1/2^: this was applied here.

**Figure 1:**
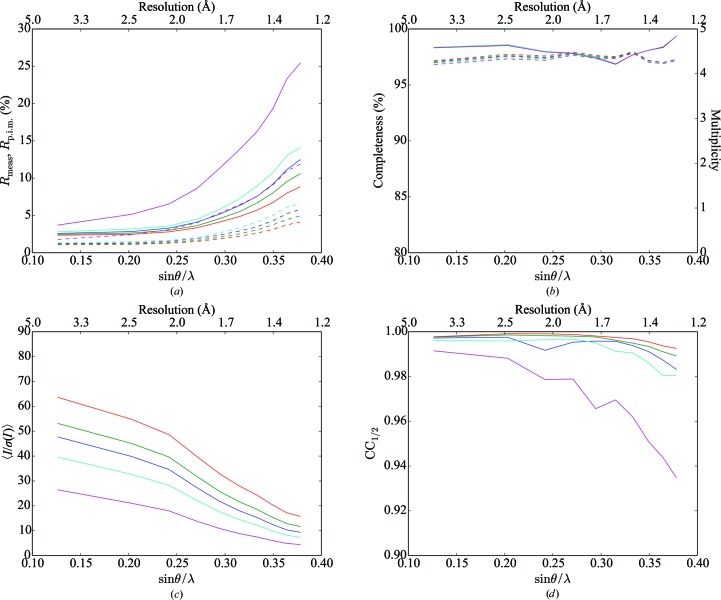
Merging statistics, including overlapping reflections, as a function of resolution for (*a*) *R*_meas_ (solid lines) and *R*_p.i.m._ (dashed lines), (*b*) completeness (solid lines) and multiplicity (dashed lines), (*c*) mean intensity over sigma and (*d*) CC_1/2_ as a function of resolution. Red, green, blue, cyan and magenta lines represent individual sweeps, two lattices, three lattices, four lattices and six lattices, respectively. Points on the abscissa represent the centre of each resolution shell.

**Figure 3:**
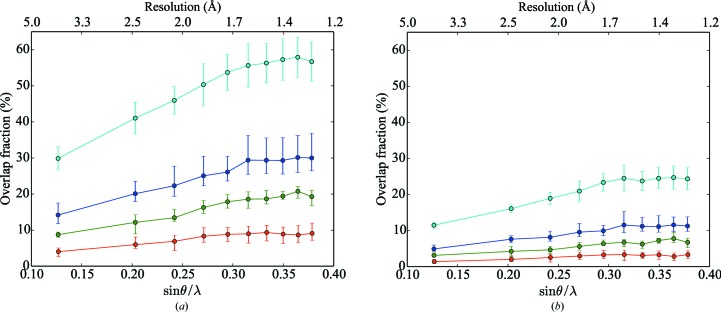
Fraction of overlaps as a function of resolution for two (red), three (green), four (blue) and six (cyan) crystals. Solid lines represent the mean values for the resolution shells; the error bars represent the minimum and maximum values in each resolution shell. Overlap fractions calculated using (*a*) *N*_σ_ = 3 and (*b*) *N*_σ_ = 2, where *N*_σ_ is the number of standard deviations used to calculate the reflection mask.

Data were integrated to the corners of the detectors (∼1.0 Å), but the resolution was truncated at 1.3 Å (the resolution of the inscribed circle on the detector) for subsequent analysis. Scaling was performed both with and without the identification of overlapping reflections; data-reduction statistics are presented in Tables 1 ▶ and 2 ▶. Figs. 1 ▶ and 2 ▶ show data-reduction statistics as a function of resolution, whilst Fig. 3 ▶ shows the fraction of overlapping spots as a function of resolution. Data-reduction statistics were calculated using *phenix.merging_statistics* (Adams *et al.*, 2010 ▶).

It is important to note that as a result of combining images from different orientations to create semi-synthetic multi-lattice data sets, the background will be between two and six times higher for the multi-lattice data sets than for the original single-lattice data sets. This is reflected in the standard deviation correction parameters applied to the measurements by *AIMLESS*, which had a relatively wide range of values for the 12 individual data sets *a*–*l*: SdFac from 0.49 to 0.79, SdB from −2.55 to 8.95 and SdAdd from 0.0277 to 0.0798. These were generally increased when determined for the six-lattice data, although the interpretation of the values is complicated by the interdependence of the parameters. The change in the mean values for these parameters from single lattice to six- lattice processing was SdFac increasing from around 0.57 to 0.75, SdB decreasing slightly from 3.04 to 2.84 and SdAdd remaining constant at around 0.047. The increase in SdFac explains, at least in part, the reduction in mean *I*/σ(*I*) as the number of lattices increases in Tables 1 ▶ and 2 ▶. In reality, for a true multi-lattice data set the background will be similar, or potentially even lower if the extra crystals displace solvent in the beam.

#### 4.1.1. Overlapping reflections   

One potential concern when faced with the presence of multiple lattices is the effect of overlapping reflections on the quality of the reduced data. In order to address this concern, we examined the fraction of overlapping reflections as a function of resolution (Fig. 3 ▶) using a value of *N*_σ_ = 3, where *N*_σ_ is the number of standard deviations used to calculate the reflection mask (§[Sec sec3.7]3.7). In contrast to Paithankar *et al.* (2011 ▶), we observe that the overlap fraction increases with resolution, which we attribute to the more sophisticated identification of integrated pixels based on the *XDS* profile model in comparison to the fixed reflection spot size assumed by Paithankar and coworkers.

Similarly to Buts *et al.* (2004 ▶), we observe that excluding overlapping reflections from scaling dramatically reduces the number of observations rejected as outliers (Tables 1 ▶ and 2 ▶). We note that there is an improvement in several data-quality indicators [in particular *R*_meas_, *R*_p.i.m._ and 〈*I*/σ(*I*)〉] when excluding overlapping reflections from scaling, at the cost of a reduction in completeness and multiplicity (Tables 1 ▶ and 2 ▶ and Figs. 1 ▶ and 2 ▶).

**Figure 4:**
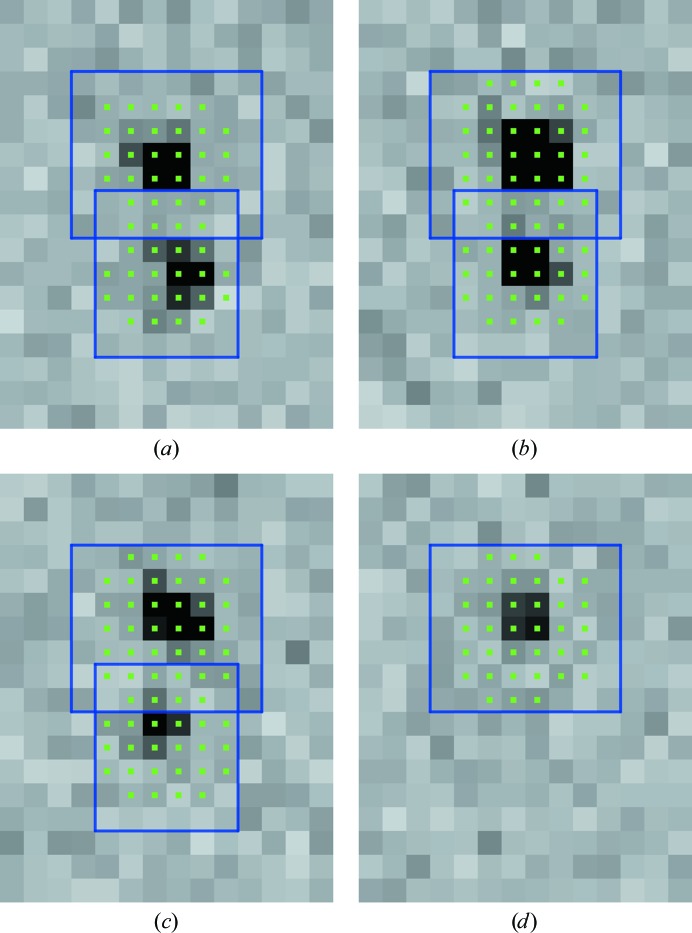
Two overlapping reflections from a semi-synthetic multi-lattice trypsin data set on four consecutive rotation images. Blue squares represent the bounding box in image space of a reflection. Green dots indicate pixels that are part of the peak region according to the values of σ_m_ and σ_b_ obtained from *XDS* (*N*_σ_ = 3). Images were generated using *dials.image_viewer*, which is derived from *cctbx.image_viewer* (Sauter *et al.*, 2013 ▶).

**Figure 5:**
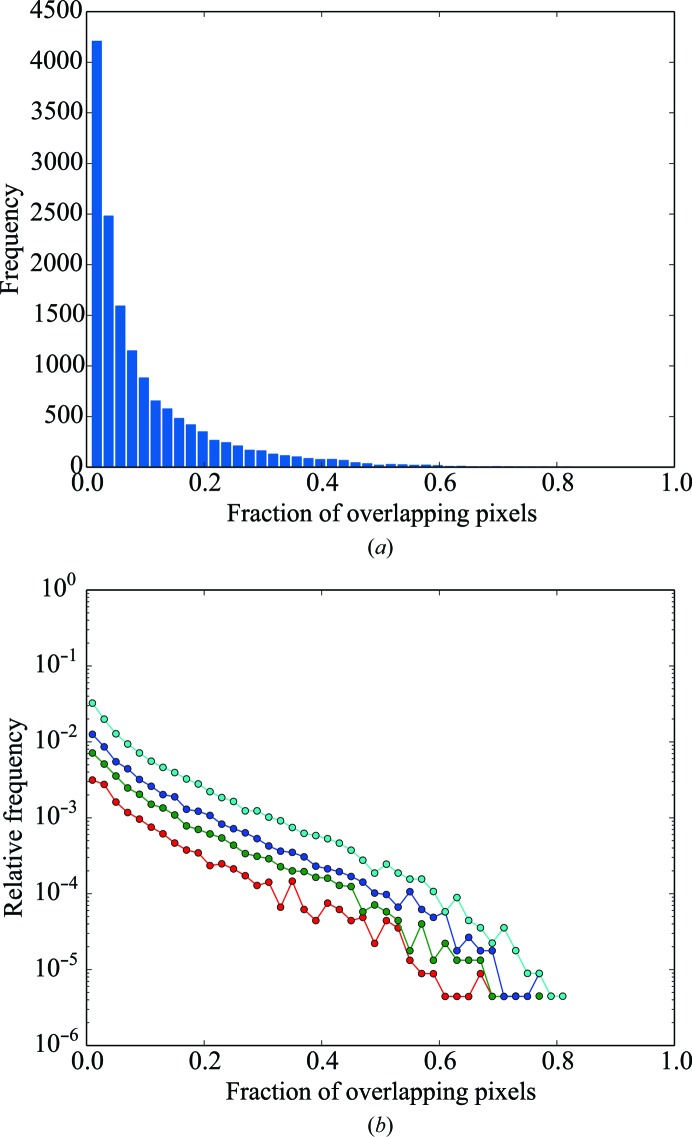
(*a*) Histogram of the fraction of overlapping pixels for the semi-synthetic six-lattice trypsin data set; (*b*) as for (*a*) but averaged across all data sets with the same number of lattices for two (red), three (green), four (blue) and six (cyan) crystals.

**Figure 6:**
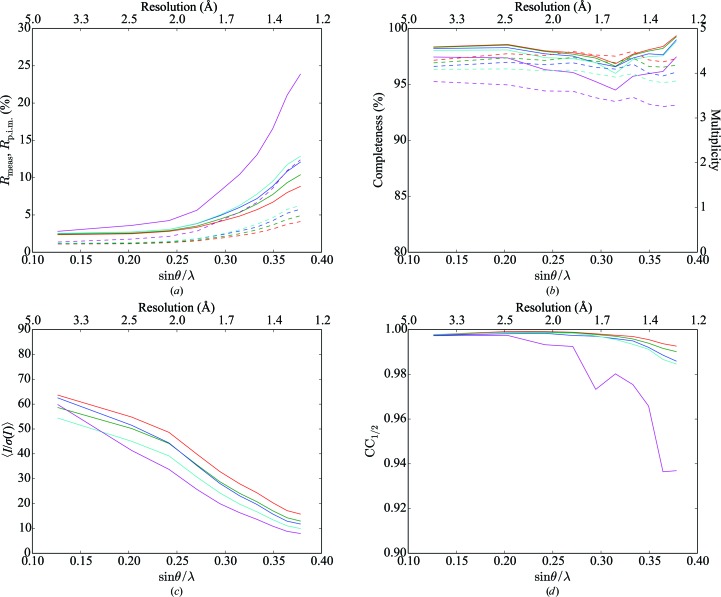
Merging statistics, excluding overlapping reflections, as a function of resolution for (*a*) *R*_meas_ (solid lines) and *R*_p.i.m._ (dashed lines), (*b*) completeness (solid lines) and multiplicity (dashed lines), (*c*) mean intensity over sigma and (*d*) CC_1/2_ as a function of resolution. Overlapping reflections calculated using *N*_σ_ = 2, where *N*_σ_ is the number of standard deviations used to calculate the reflection mask. Red, green, blue, cyan and magenta lines represent individual sweeps, two crystals, three crystals, four crystals and six crystals, respectively. Points on the abscissa represent the centre of each resolution shell.

Inspection of the diffraction images suggests qualitatively that many of the overlaps identified through the procedure described above in fact only involve the overlap of a few pixels from each reflection (see, for example, Fig. 4 ▶). This was confirmed by a histogram of the fraction of overlapping pixels (Fig. 5 ▶), indicating that the majority of overlapping reflections overlap only in the tails of the peak region. This suggests that reducing the number of standard deviations, *N*_σ_, used to calculate the reflection mask profiles would ensure that only pairs of reflections that are overlapping in the central peak region are rejected, with minimal impact on data quality. Scaling was repeated excluding overlapping reflections calculated using *N*_σ_ = 2. The resulting merging statistics were similar to those obtained using *N*_σ_ = 3, but with higher values of completeness and multiplicity, particularly for the six-lattice data set (Table 3 ▶ and Fig. 6 ▶). The choice of an optimal value of *N*_σ_ to be used in the identification of overlapping reflections is likely to involve a compromise between data quality and completeness. A more advanced approach would require the modification of integration software such that it is aware of the presence of multiple lattices, enabling the exclusion from background determination and profile fitting of pixels belonging to overlapping reflections (Fry *et al.*, 1993 ▶). Alternatively, peak deconvolution procedures during integration such as those described by Bourgeois *et al.* (1998 ▶) or Schreurs *et al.* (2010 ▶) may work well in such cases.

#### 4.1.2. Very narrow wedges   

It is widely recognized that the robustness of current indexing algorithms can be increased by using data from images that are widely separated in reciprocal space (for example, separated by a rotation of 90°), particularly for more problematic cases (Steller *et al.*, 1997 ▶; Sauter *et al.*, 2004 ▶; Powell *et al.*, 2013 ▶; Winter *et al.*, 2013 ▶). Therefore, this can make the indexing of multiple lattices from narrow wedges of data (*e.g.* 1° rotation images or XFEL still shots) especially challenging.

In order to further test our multi-lattice indexing algorithm, we ran *dials.index* using just the first 1° of images for the semi-synthetic multi-lattice data sets described above. In all cases, from the two-lattice data sets to the six-lattice data sets, all 12 lattices were successfully identified and the crystal orientation refined to within less than 0.05° of the orientation obtained from the full 10° of single-lattice data.

We then tested the performance of the algorithm using just the first image from each sweep and found that all six lattices were successfully identified from a single image of each six-lattice data set. This result demonstrates the applicability of the algorithm to very narrow wedges of data and potentially also to XFEL data, where the nature of the current sample-delivery systems can result in multiple lattices being present in the beam simultaneously (Hattne *et al.*, 2014 ▶; Sawaya *et al.*, 2014 ▶).

#### 4.1.3. Comparison to existing methods   

In order to assess the performance of our methods in comparison to existing methods, indexing was attempted with the recent implementation of multi-lattice indexing in *iMosflm* (Powell *et al.*, 2013 ▶). When provided with the first 1° of images from the six-lattice data sets, *iMosflm* identified five lattices (only two of which had the correct unit cell) for one of the data sets and five lattices (only one of which had the correct unit cell) for the second data set. When indexing was attempted using only the first image of each data set, *iMosflm* identified four lattices (of which three had the correct unit cell) for the first data set and only one lattice (with an incorrect unit cell) for the second data set.

Whilst *XDS* itself does not implement multi-lattice indexing, it is possible to extract the list of unindexed spots from the output of *XDS* indexing and use these as input to a subsequent run of the *IDXREF* step in order to attempt indexing of a further lattice (http://strucbio.biologie.uni-konstanz.de/xdswiki/index.php/Indexing). Using this approach, we attempted indexing with *XDS* using just the first image of each of the six-lattice data sets. For one of the data sets (*acegik*) *XDS* was able to successfully identify all six lattices. However, for the other data set (*bdfhjl*) *XDS* apparently identified five lattices, but on further inspection an incorrect unit cell was chosen by *XDS* in spite of the known unit cell and symmetry being provided as input.

Whilst this is not intended to be a rigorous comparison of the robustness of different indexing algorithms and implementations, it serves to demonstrate that the availability of a variety of algorithms can be beneficial to the user community as each algorithm has a its own set of strengths and weaknesses.

### 4.2. Polyhedra microcrystal data   

Polyhedra are naturally formed protein microcrystals produced by cypoviruses and baculoviruses, in which virus particles are embedded as part of an infectious cycle targeting insects (Chiu *et al.*, 2012 ▶). The polyhedra protect the virus particles against hostile conditions and allow them to survive for long periods prior to ingestion and particle release within the insect gut. These crystals typically only grow within the insect cells to a few micrometres in size (with the maximum size depending on the virus species). Early synchrotron studies used powder diffraction to show that although their biological structure varies substantially, with little similarity in their amino-acid sequences, they form virtually identical crystal lattices in space group *I*23 with very similar unit-cell dimensions (*a* ≃ 100 Å; Anduleit *et al.*, 2005 ▶). Recent studies have successfully used microfocus beamlines at third-generation synchrotron sources to obtain molecular structures from single crystals (Coulibaly *et al.*, 2007 ▶, 2009 ▶; Ji *et al.*, 2010 ▶). The crystals studied to date have typically been on the order of 5–12 µm, but Axford *et al.* (2014 ▶) have recently reported high-quality data obtained from crystals of only 4–5 µm in size.

**Figure 7:**
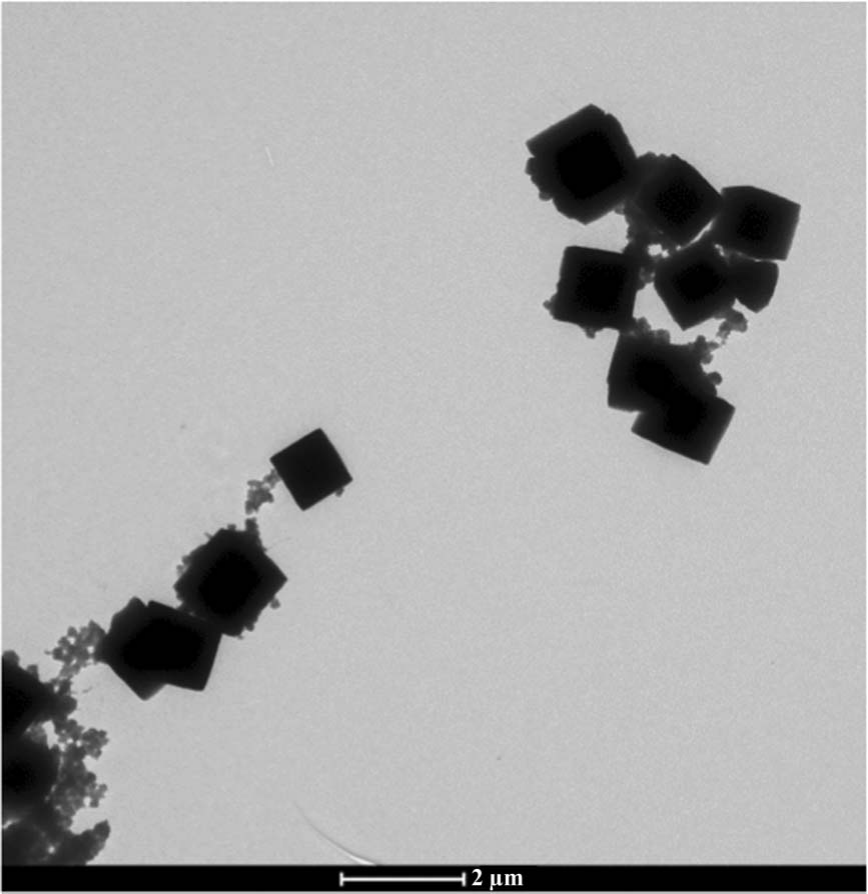
A TEM image of polyhedrin crystals from a polyhedrosis virus as used in §[Sec sec4.2]4.2. Typical crystal size is <1 µm.

**Figure 8:**
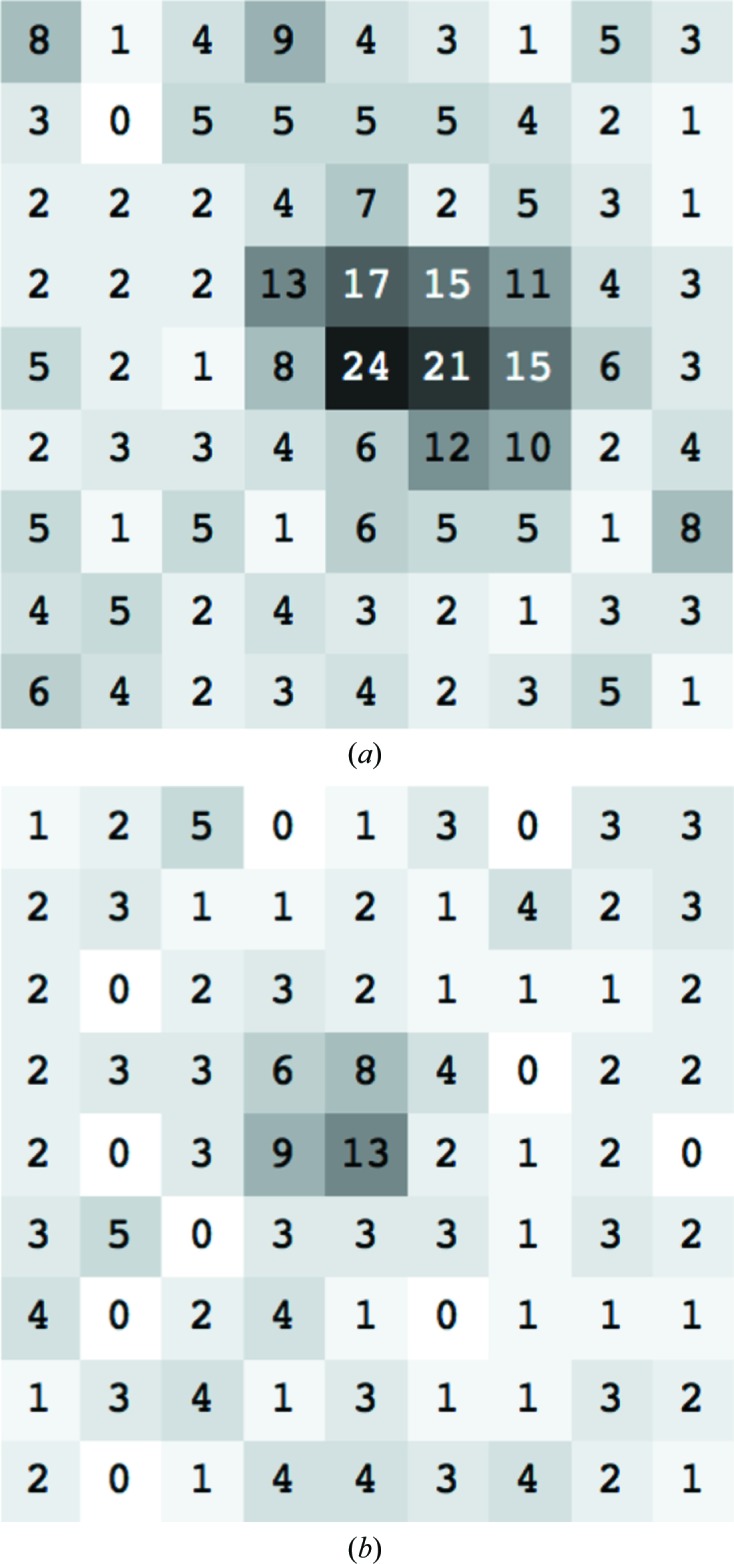
An illustration of the strength of diffracted intensities and spot size for the crystals in §[Sec sec4.2]4.2: the intensities of the pixels surrounding spots whose total intensities (using simple summation of raw pixel counts) are at the 90th percentile (*a*) and the 10th percentile (*b*), based on spots identified by *dials.find_spots* for a single sweep of data. Images were prepared using *phenix.image_viewer* (Echols *et al.*, 2012 ▶).

Data for a previously unstudied polyhedrin were collected on the I24 beamline at Diamond Light Source from crystals on the order of 1 µm in size (Fig. 7 ▶) using an X-ray beam with a cross-section of ∼4 × 4 µm at the sample. Individual crystals could not be resolved with the beamline on-axis viewing system; therefore, data were collected at locations identified using grid scans (Aishima *et al.*, 2010 ▶) with the help of the *DISTL* software (Zhang *et al.*, 2006 ▶). Diffraction was extremely weak (Fig. 8 ▶) and therefore required very long exposures per frame, and as a result only 1° of data could be collected per crystal. 420 data sets (20 × 0.05° images) were collected, but automated data processing using the *XDS* pipeline within *xia*2 proved problematic, with few data sets processing successfully (160 out of 420).

**Figure 9:**
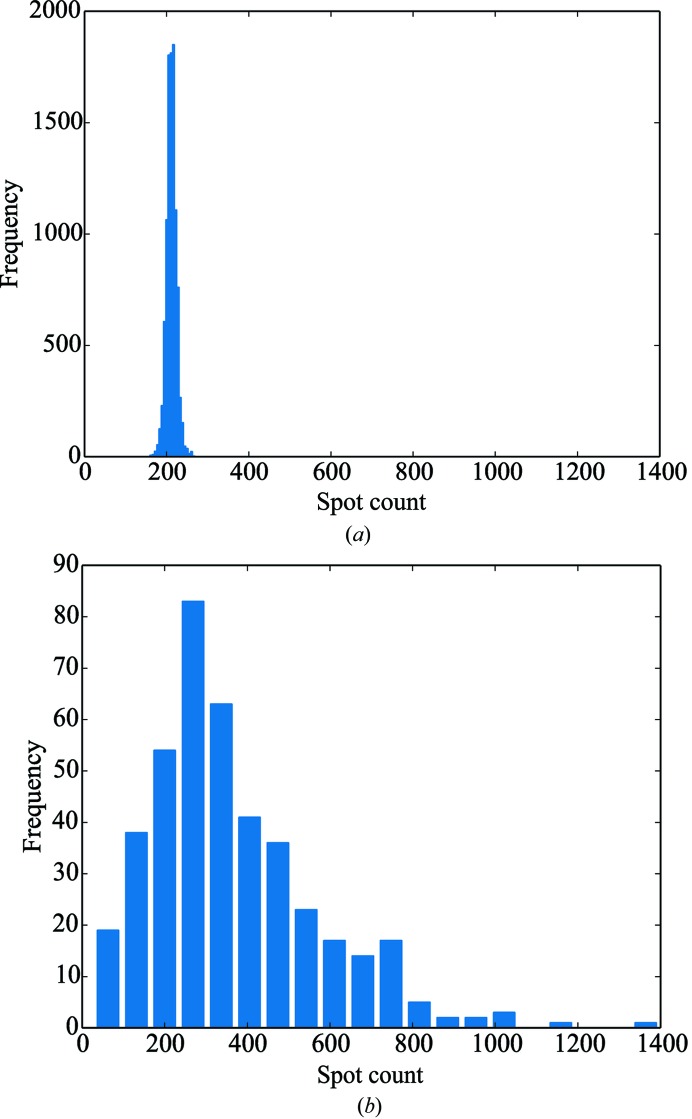
An excess number of observed spots over those predicted suggests the presence of multiple lattices: (*a*) histogram of the number of predicted centroids to a resolution of 4 Å per 1° wedge of data for 10 000 random orientations, (*b*) histogram of the number of spots found in §[Sec sec4.2]4.2 to a resolution of 4 Å per 1° wedge of data.

**Figure 10:**
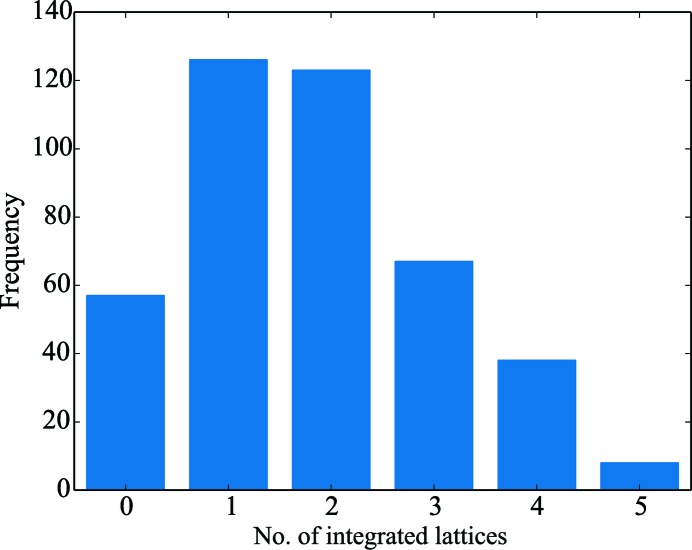
Histogram of the number of successfully integrated lattices per sweep for the data in §[Sec sec4.2]4.2.

**Figure 11:**
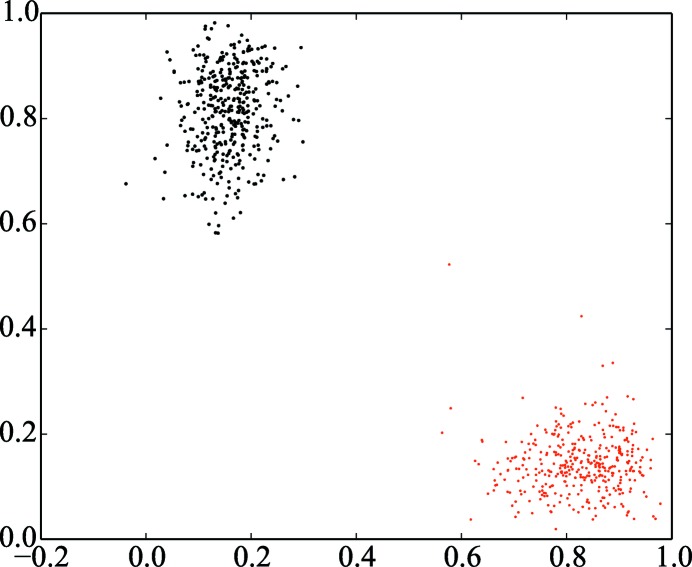
Application of algorithm 2 of Brehm & Diederichs (2014 ▶) to the data in §[Sec sec4.2]4.2. Points are coloured according to the assigned indexing mode. Identification and rejection prior to scaling of sweeps that have poor correlation with either indexing mode may improve the quality of the final merged data set.

Analysis of the number of spots per data set found using *dials.find_spots* compared with the number expected based on the unit-cell dimensions gave a clear indication of the presence of multiple lattices (Fig. 9 ▶). *dials.index* was used in multi-lattice mode on the output of *dials.find_spots*, identifying a total of 997 lattices, of which 768 were integrated successfully with *XDS*, representing a significant improvement compared with that obtained using *XDS* *via xia*2. The majority of sweeps were found to have more than one lattice present, with up to five lattices successfully integrated in some cases (Fig. 10 ▶). The space group *I*23 can be indexed in one of two ways; hence, it was necessary to ensure that all lattices were indexed in a consistent manner. This was achieved using the algorithm of Brehm & Diederichs (2014 ▶) as described in §[Sec sec3.8]3.8, which showed a clear separation of the two indexing modes (Fig. 11 ▶).

### 4.3. Applications   

#### 4.3.1. *xia*2   

While the algorithms described above are useful in isolation, when faced with data sets consisting of many tens or even hundreds of sweeps some level of automation becomes critical. The *dials.index* tool and associated spot-finding and refinement commands have been incorporated into *xia2* (Winter, 2010 ▶) and used with *XDS* for integration. While this works well for data sets with single lattices visible on the images, the design of *xia*2 is such that processing multiple lattices is currently not possible: for sweeps with multiple lattices only the first lattice identified will be processed. Work is ongoing to redesign this aspect of *xia*2 to offer the user an automated tool for processing multi-lattice data.

#### 4.3.2. Diamond Light Source   

As these algorithms are not yet fully integrated into *xia2*, they have been added to the automatic processing scripts that are running following data collection on Diamond MX beamlines (Winter & McAuley, 2011 ▶) to provide the user with feedback about (i) whether the data can be indexed and (ii) the number of lattices present. While the former of these may be used to provide data *via XDS* to *BLEND* (Foadi *et al.*, 2013 ▶), the latter provides useful feedback on the sample density and can guide subsequent sample preparation.

## 5. Availability   

*DIALS* is available for download from http://sourceforge.net/projects/dials and the source code is available under a non­restrictive open-source BSD license. The program *dials.index* also includes an implementation of the one-dimensional FFT indexing methods of Steller *et al.* (1997 ▶) derived from the open-source components of *LABELIT* (Sauter *et al.*, 2004 ▶) and an implementation of three-dimensional FFT indexing methods (Bricogne, 1986 ▶; Campbell, 1998 ▶), both of which do not require prior knowledge of the unit cell.

The original trypsin images and the semi-synthetic multi-lattice images are publicly available at http://zenodo.org/record/10820 (Gildea & Winter, 2014 ▶).

## 6. Conclusions   

New indexing algorithms have been presented which aid the analysis of microcrystal X-ray diffraction data by overcoming some of the key indexing challenges, namely handling narrow sweeps of data containing spots from multiple crystal lattices. These algorithms have been developed within the *DIALS* framework but may be applied with other integration software such as *XDS*. In dealing with experimental data where multiple lattices are present it was demonstrated that the treatment of overlapping peaks was necessary to obtain good-quality data; however, doing so required the development of additional tools within the *DIALS* framework. Given the similarities between the serial crystallography discussed here and XFEL data collection, it is only fitting that the algorithms may be shared: we anticipate that the indexing algorithms presented here may be equally applicable to XFEL data. 

## Figures and Tables

**Figure 2:**
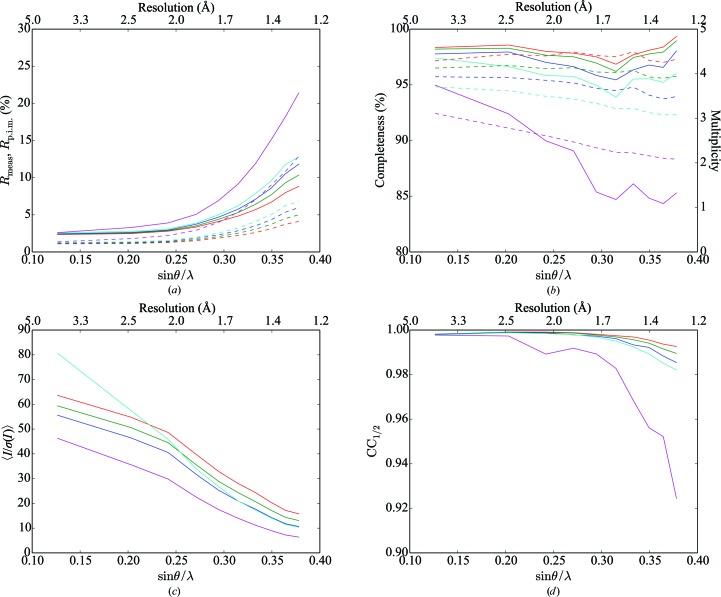
Merging statistics, excluding overlapping reflections, as a function of resolution for (*a*) *R*_meas_ (solid lines) and *R*_p.i.m._ (dashed lines), (*b*) completeness (solid lines) and multiplicity (dashed lines), (*c*) mean intensity over sigma and (*d*) CC_1/2_ as a function of resolution. Overlapping reflections calculated using *N*_σ_ = 3, where *N*_σ_ is the number of standard deviations used to calculate the reflection mask. Red, green, blue, cyan and magenta lines represent individual sweeps, two crystals, three crystals, four crystals and six crystals, respectively. Points on the abscissa represent the centre of each resolution shell.

**Table 1:** Data-reduction statistics for the semi-synthetic multi-lattice data sets, including overlapping reflections Values in parentheses are for the outer resolution shell. *R*_meas_, *R*_p.i.m._ and CC_1/2_ are calculated as defined by Diederichs & Karplus (1997 ▶), Weiss (2001 ▶) and Karplus & Diederichs (2012 ▶), respectively. The number of rejected reflections refers to the reflections identified as outliers during scaling.

Data set	12 × one-lattice	6 × two-lattice	4 × three-lattice	3 × four-lattice	2 × six-lattice
Resolution range (Å)	43.85–1.30 (1.35–1.30)	43.85–1.30 (1.35–1.30)	43.85–1.30 (1.35–1.30)	43.85–1.30 (1.35–1.30)	43.87–1.30 (1.35–1.30)
No. of reflections: total/unique	225096/51437	224218/51438	223517/51438	222939/51432	222303/51514
No. of rejected reflections	70	832	1352	1733	1977
Completeness (%)	98.1 (99.3)	98.1 (99.3)	98.1 (99.3)	98.0 (99.3)	98.0 (99.4)
Multiplicity	4.4 (4.3)	4.4 (4.3)	4.3 (4.3)	4.3 (4.3)	4.3 (4.3)
*R*_meas_ (%)	3.0 (8.8)	3.3 (10.6)	3.6 (12.5)	4.1 (14.1)	6.9 (25.4)
*R*_p.i.m._ (%)	1.4 (4.1)	1.5 (4.9)	1.7 (5.8)	1.9 (6.6)	3.3 (11.9)
〈*I*/σ(*I*)〉	34.8 (15.8)	27.8 (11.7)	23.9 (9.3)	19.4 (7.4)	12.3 (4.4)
CC_1/2_ (%)	99.8 (99.3)	99.8 (98.9)	99.8 (98.3)	99.7 (98.1)	99.3 (93.5)

**Table 2:** Data-reduction statistics for the semi-synthetic multi-lattice data sets, excluding overlapping reflections prior to scaling, using *N*_σ_ = 3, where *N*_σ_ is the number of standard deviations used to calculate the reflection mask Values in parentheses are for the outer resolution shell.

Data set	12 × one-lattice	6 × two-lattice	4 × three-lattice	3 × four-lattice	2 × six-lattice
Resolution range Å)	43.85–1.30 (1.35–1.30)	43.85–1.30 (1.35–1.30)	43.85–1.30 (1.35–1.30)	43.85–1.30 (1.35–1.30)	43.87–1.30 (1.35–1.30)
No. of reflections: total/unique	225096/51437	207352/51242	187700/50804	167348/50193	111993/46119
No. of rejected reflections	70	75	177	162	130
Fraction of overlaps (%)	–	6.3–9.4	15.0–18.0	22.7–30.9	45.3–55.1
Completeness (%)	98.1 (99.3)	97.7 (99.0)	96.8 (98.0)	95.7 (96.0)	87.8 (85.3)
Multiplicity	4.4 (4.3)	4.0 (3.9)	3.7 (3.5)	3.3 (3.1)	2.4 (2.1)
*R*_meas_ (%)	3.0 (8.8)	3.2 (10.4)	3.3 (11.8)	3.5 (12.8)	4.0 (21.4)
*R*_p.i.m._ (%)	1.4 (4.1)	1.5 (5.0)	1.6 (6.0)	1.8 (6.8)	2.2 (12.9)
〈*I*/σ(*I*)〉	34.8 (15.8)	31.1 (13.0)	27.8 (10.6)	32.4 (10.3)	20.7 (6.4)
CC_1/2_ (%)	99.8 (99.3)	99.9 (99.0)	99.9 (98.5)	99.9 (98.2)	99.8 (92.4)

**Table 3:** Data-reduction statistics for the semi-synthetic multi-lattice data sets, excluding overlapping reflections prior to scaling, using *N*_σ_ = 2, where *N*_σ_ is the number of standard deviations used to calculate the reflection mask Values in parentheses are for the outer resolution shell.

Data set	12 × one-lattice	6 × two-lattice	4 × three-lattice	3 × four-lattice	2 × six-lattice
Resolution range (Å)	43.85–1.30 (1.35–1.30)	43.85–1.30 (1.35–1.30)	43.85–1.30 (1.35–1.30)	43.85–1.30 (1.35–1.30)	43.87–1.30 (1.35–1.30)
No. of reflections: total/unique	225096/51437	218529/51377	211277/51253	202828/51134	176631/50568
No. of rejected reflections	70	192	439	289	575
Fraction of overlaps (%)	—	2.1–3.4	5.4–6.2	8.5–11.7	18.8–23.4
Completeness (%)	98.1 (99.3)	97.9 (99.2)	97.7 (99.0)	97.5 (98.8)	96.2 (97.4)
Multiplicity	4.4 (4.3)	4.3 (4.2)	4.1 (4.0)	4.0 (3.8)	3.5 (3.3)
*R*_meas_ (%)	3.0 (8.8)	3.2 (10.4)	3.5 (12.1)	3.5 (12.9)	4.9 (23.9)
*R*_p.i.m._ (%)	1.4 (4.1)	1.5 (4.9)	1.6 (5.8)	1.7 (6.3)	2.5 (12.4)
〈*I*/σ(*I*)〉	34.8 (15.8)	30.9 (13.0)	30.8 (11.8)	26.7 (9.9)	24.2 (8.0)
CC_1/2_ (%)	99.8 (99.3)	99.9 (99.0)	99.8 (98.6)	99.9 (98.5)	99.8 (93.7)

## Appendix A

### A1. Coordinate frames

#### A1.1. The diffractometer equation

We use the vector **h** to describe a position in fractional reciprocal space in terms of the reciprocal-lattice basis vectors **a***, **b*** and **c***,

The special positions at which *h*, *k* and *l* are integer define the reciprocal-lattice vectors for which (*hkl*) are the Miller indices.

The basic diffractometer equation relates a position **h** to a position **r**_ϕ_ in Cartesian reciprocal space. This space is defined so that its axes coincide with the axes of the laboratory frame. The distinction is necessary because distances in reciprocal space are measured in units of Å^−1^. However, for convenience it is often acceptable to refer to either Cartesian reciprocal space or the real-space laboratory frame as the ‘laboratory frame’, when the correct choice is clear by context. The diffractometer equation is

where **R** is the goniostat rotation matrix and **A** is the crystal setting matrix, while its inverse **A** ^−1^ is referred to as the indexing matrix. The product **Ah** may be written as **r**, which is a position in the ϕ-axis frame, a Cartesian frame that coincides with the laboratory frame at a rotation angle of ϕ = 0. This makes clear that the setting matrix does not change during the course of a rotation experiment (notwithstanding small ‘mis-set’ rotations).

For an experiment performed using the rotation method, we use ϕ to refer to the angle about the actual axis of rotation, even when this is effected by a differently labelled axis on the sample-positioning equipment (such as an ω axis of a multi-axis goniometer).

### A2. Orthogonalization convention

Following Busing & Levy (1967 ▶), we may decompose the setting matrix **A** into the product of two matrices, conventionally labelled **U** and **B**. We name **U** the orientation matrix and **B** the reciprocal-space orthogonalization matrix. These names are in common, but not universal, use. In particular, some texts (for example, Paciorek *et al.*, 1999 ▶) refer to the product (*i.e.* our setting matrix) as the ‘orientation matrix’.

Of these two matrices, **U** is a pure rotation matrix and is dependent on the definition of the laboratory frame, whilst **B** is not dependent on this definition. **B** does depend, however, on a choice of orthogonalization convention, which relates **h** to a position in the crystal-fixed Cartesian system. The basis vectors of this orthogonal Cartesian frame are fixed to the reciprocal lattice *via* this convention.

Although there is no single unique way that **A** may be decomposed into a pair **UB**, it is always possible to extract the unit-cell dimensions irrespective of the orthogonalization conventions, since **A** ^T^ **A** = **B** ^T^ **B** , which is the reciprocal metric matrix. The symbolic expression of **B** is simplest when the crystal-fixed Cartesian system is chosen to be aligned with the crystal real-space or reciprocal-space axes. For example, Busing & Levy (1967 ▶) use a frame in which the basis vector **i** is parallel to reciprocal-lattice vector **a***, while **j** is chosen to lie in the plane of **a*** and **b***. Unfortunately, this convention is then disconnected from the standard real-space orthogonalization convention, usually called the PDB convention (Protein Data Bank, 1992 ▶). This standard is essentially universal in crystallographic software for the transformation of fractional crystallographic coordinates to positions in orthogonal space, with units of Å. In particular, it is the convention used in *cctbx* (Grosse-Kunstleve *et al.*, 2002 ▶). The convention states that the orthogonal coordinate *x* is determined from a fractional coordinate *u* by

where the matrix **O** is the real-space orthogonalization matrix. This matrix transforms to a crystal-fixed Cartesian frame that is defined such that its basis vector **i** is parallel to the real-space lattice vector **a**, while **j** lies in the (**a**, **b**) plane. The elements of this matrix made explicit in a compact form are

It is desirable to specify our reciprocal-space orthogonalization convention in terms of this real-space orthogonalization convention. Giacovazzo (2002 ▶) derives relationships between real and reciprocal space. Of particular interest from that text, we have

By analogy, equate **x***′ with **h** and **B** with **M** ^−1^. Also equate **M** ^T^ with **O** and **x**′ with **u**. We then see that

where **F** is designated the real-space fractionalization matrix.

### A3. Orientation matrix

The matrix **U** ‘corrects’ for the orthogonalization convention implicit in the choice of **B**. As the crystal-fixed Cartesian system and the ϕ-axis frame are both orthonormal Cartesian frames with the same scale, it is clear that **U** must be a pure rotation matrix. Its elements are clearly dependent on the mutual orientation of these frames.
